# The complementary value of intraoperative fluorescence imaging and Raman spectroscopy for cancer surgery: combining the incompatibles

**DOI:** 10.1007/s00259-022-05705-z

**Published:** 2022-02-01

**Authors:** L. J. Lauwerends, H. Abbasi, T. C. Bakker Schut, P. B. A. A. Van Driel, J. A. U. Hardillo, I. P. Santos, E. M. Barroso, S. Koljenović, A. L. Vahrmeijer, R. J. Baatenburg de Jong, G. J. Puppels, S. Keereweer

**Affiliations:** 1 Department of Otorhinolaryngology, Head and Neck Surgery, Erasmus MC Cancer Institute, Rotterdam, Netherlands; 2 Center for Optical Diagnostics and Therapy, Department of Dermatology, Erasmus MC Cancer Institute, Rotterdam, Netherlands; 3 Department of Orthopedic Surgery, Isala Hospital, Zwolle, Netherlands; 4 Molecular Physical-Chemistry R&D Unit, Department of Chemistry, University of Coimbra, Coimbra, Portugal; 5 AlfaRim Medical Holding, Delft, Netherlands; 6 Department of Pathology, Antwerp University Hospital/Antwerp University, Antwerp, Belgium; 7 Department of Surgery, Leiden University Medical Center, Leiden, Netherlands

**Keywords:** Raman spectroscopy, Fluorescence imaging, Image-guided surgery, Tumour differentiation, Resection margin assessment, Multimodal optical diagnostics

## Abstract

A clear margin is an important prognostic factor for most solid tumours treated by surgery. Intraoperative fluorescence imaging using exogenous tumour-specific fluorescent agents has shown particular benefit in improving complete resection of tumour tissue. However, signal processing for fluorescence imaging is complex, and fluorescence signal intensity does not always perfectly correlate with tumour location. Raman spectroscopy has the capacity to accurately differentiate between malignant and healthy tissue based on their molecular composition. In Raman spectroscopy, specificity is uniquely high, but signal intensity is weak and Raman measurements are mainly performed in a point-wise manner on microscopic tissue volumes, making whole-field assessment temporally unfeasible. In this review, we describe the state-of-the-art of both optical techniques, paying special attention to the combined intraoperative application of fluorescence imaging and Raman spectroscopy in current clinical research. We demonstrate how these techniques are complementary and address the technical challenges that have traditionally led them to be considered mutually exclusive for clinical implementation. Finally, we present a novel strategy that exploits the optimal characteristics of both modalities to facilitate resection with clear surgical margins.

## Introduction

Cancer is the leading cause of premature death in Europe and the USA and one of the most important public health issues worldwide [1, 2]. Depending on the tumour type and stage, different treatment options are available. Surgery is the main treatment modality for most solid tumours, often integrated in a more extensive (neo-)adjuvant treatment strategy. Surgical resection is generally aimed at complete macroscopic and microscopic removal of the cancer. However, inadequate resection of the tumour (i.e. presence of cancer cells at the margin or < 5 mm from the resection surface) frequently occurs. The worldwide extent of this problem is illustrated in Table 1, showing the percentage of positive surgical margins for the most common cancer types. Although a clear surgical margin may not be pivotal in all cancer types (e.g. during debulking procedures for brain tumours [3]), it is the main prognostic factor for survival in most types of cancer. Moreover, inadequate surgical tumour resection most often warrants adjuvant treatment, which is associated with increased morbidity and costs. During the operation, an oncologic surgeon takes into account all relevant clinical information that is available, including cancer type, tumour differentiation, and preoperative imaging reports. However, the actual surgical cut is based on subjective tactile and visual assessment of the tissue to determine which tissue needs to be excised. Although the primary aim is to completely excise the tumour, precise margin delineation is imperative in many delicate areas where wider resections inevitably lead to increased morbidity and loss of functionality.

**Table 1:** Percentage of positive surgical margins for the most common cancer types (estimated new cases worldwide, 2020). *Males and females combined

Cancer type (solid tumours only)	Estimated new cases [4]*	Incidence [4]*	Positive margins
Breast	2,261,419	12%	20–70% [5–8]
Trachea, lung, and bronchus	2,206,771	11%	5–17% [9–12]
Prostate	1,414,259	7%	7–75% [13–16]
Colorectal	1,931,590	10%	12–58% [17–19]
Urinary bladder	573,278	3%	0–25% [20–22]
Kidney and renal pelvis	431,288	2%	7–11% [23–26]
Uterine corpus	417,367	2%	4–17% [8, 27]
Pancreas	495,773	3%	18–85% [28–31]
Thyroid	586,202	3%	10–11% [8, 32, 33]
Lip, oral cavity	377,713	2%	5–43% [34, 35]

Over the past two decades, important advances have been made to improve assessment of surgical margins by the introduction of intraoperative imaging techniques. Besides frozen section analysis, several optical methods have been studied to perform intraoperative margin assessment in surgical oncology, including optical coherence tomography (OCT) [36], photoacoustic tomography, terahertz imaging, second harmonic generation, confocal microscopy, fluorescence (lifetime) imaging, autofluorescence imaging (AFI), narrow-band imaging, hyper-spectral imaging, diffuse reflectance spectroscopy, Fourier transform infrared spectroscopy, and Raman spectroscopy (RS) [37–42]. Among them, intraoperative fluorescence imaging (FLI) using exogenous tumour-specific fluorescent agents has shown to be particularly beneficial for surgical margin assessment in clinical trials [43–54], and it has been shown that RS can objectively discriminate between normal and malignant tissue [55–59]. Intraoperative FLI provides a wide-field real-time tumour-specific image to guide the surgical resection. However, detection and interpretation of the fluorescent signal are complex, and are influenced by optical tissue properties, targeting specificity of fluorescent agents and accuracy of the imaging system. Each of these factors has an effect on image resolution, sensitivity, and/or specificity of the technique [60]. RS on the other hand provides detailed identification of malignant tissues with high sensitivity and specificity [61]. It does not provide the surgeon with a wide-field image because it generally requires ‘point’ (i.e. of small tissue volumes) measurements of the tissue. This compromises the possibility of scanning the complete resection surface.

In this review, we describe the state-of-the-art of FLI and RS and include an overview of the advantages and limitations for their use in image-guided cancer surgery. Next, we demonstrate how these two techniques are complementary and how they can overcome each other’s disadvantages and limitations. Finally, we highlight the technical challenges that need to be addressed to implement this novel approach into clinical practice.

## Image-guided cancer surgery using fluorescence imaging

**Fig. 1:**
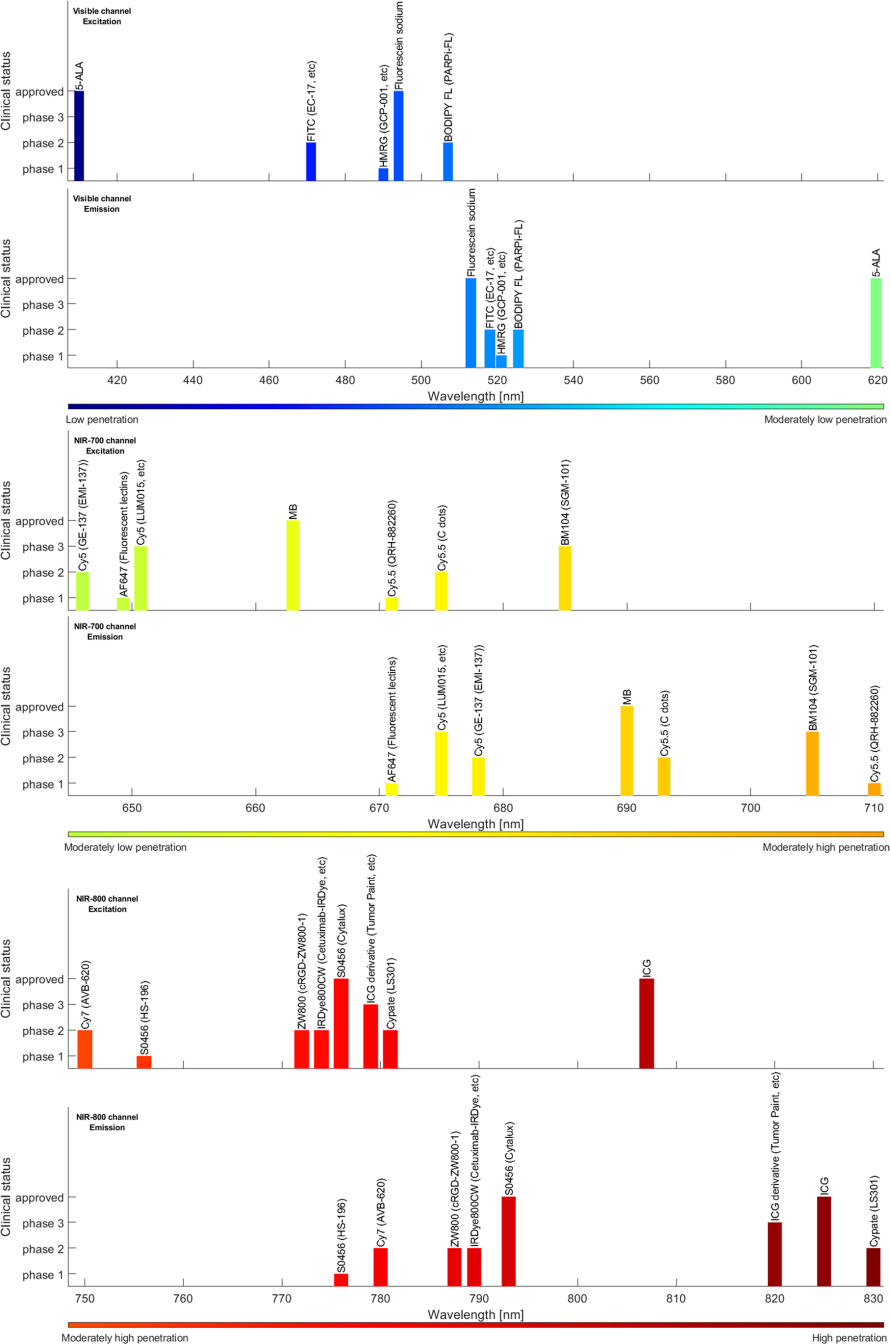
Excitation and emission wavelengths of approved and clinical fluorescent agents in the visible channel, NIR-700-nm channel, and NIR-800-nm channel (data collected from [108–112])

Intraoperative FLI of tumour tissue requires the systemic administration of a cancer-specific exogenous fluorescent agent that can identify the tumour using a spectrum of targeting strategies, including antibodies, small peptides, and activatable fluorophores [62–64]. The use of fluorophores that emit light in the first near-infrared (NIR-I) range (650–900 nm) results in deeper tissue penetration of the photons and lower autofluorescence from surrounding tissues [65–67]. Because the human eye is insensitive to light in this spectrum, dedicated intraoperative camera systems are required to detect the fluorescent signal [45, 68–71]. Figure 1 shows the clinical status of targeted fluorescent agents in the visible channel, NIR-I-700-nm channel, and NIR-I-800-nm channel together with their excitation and emission wavelengths.

FLI exhibits several favourable characteristics for clinical translation. Preclinical studies have demonstrated low toxicity with high sensitivity and specificity for tumour detection using targeted fluorescent agents [62, 63, 72, 73]. Besides the minimal risks associated with the use of lasers in the imaging system, there are no safety issues for the clinicians because no ionising radiation is used, and the technique is capable of real-time detection without interfering with the surgical field. Importantly, the whole-field imaging facilitates analysis of the complete surgical margin and remaining wound bed.

**Fig. 2:**
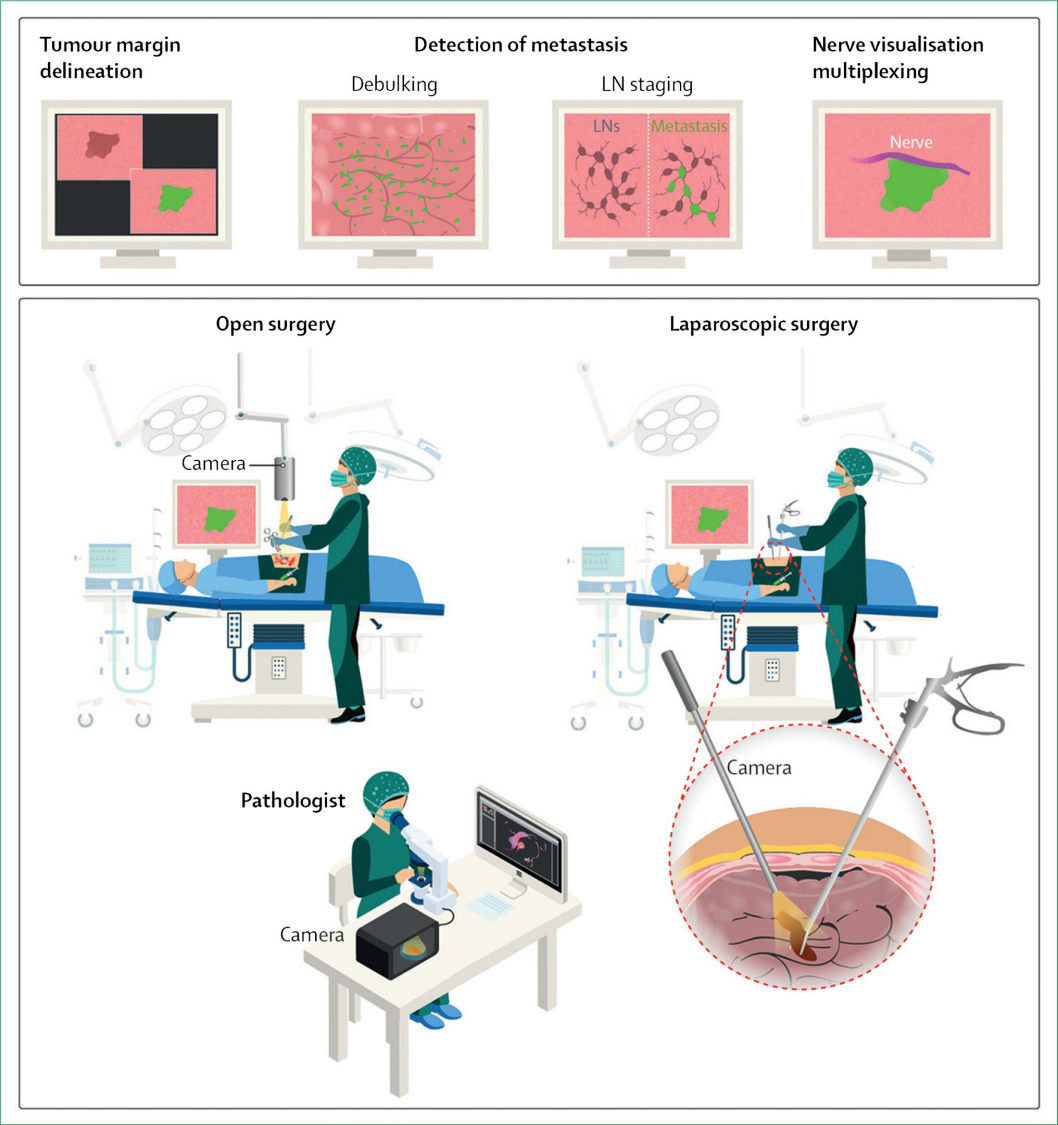
(Top) Schematic illustration of the intraoperative impact of using fluorescence-guided surgeries. (Bottom) Implementation of fluorescence guidance. Following the administration of the fluorescent agent, the tissues of interest can be visualised with a dedicated NIR camera in real-time during open or laparoscopic surgery. In addition to guiding the surgeon during the resection, rapid feedback on the presence of fluorescence can be provided by imaging the resected tissue on a back table with a NIR camera and microscope in the operating room (taken from [79] with permission)

The simultaneous development of clinically available fluorescent agents and intraoperative camera systems over the past decade has now positioned intraoperative FLI as a promising real-time detection modality for surgeons in oncology. Since the first clinical trial of FLI with tumour-specific NIR fluorescent agents [45], ground-breaking results have been reported in patients with various cancer types, consistently showing excellent safety records [43–54, 74]. Indocyanine green (ICG), a nonspecific and US Food and Drug Administration approved compound, has commonly been used for FLI in various settings [75, 76]. Methylene blue (MB) was approved as the second NIR fluorescent agent for fluorescence-based intraoperative imaging [77]. Fluorescein sodium, 5-ALA, and Cytalux/Pafolacianine are three other clinically approved fluorescent agents. To date, fluorescent agents [78, 79] have been developed for detection of cancer cells [80–83], sentinel lymph nodes [43, 71, 84, 85], atherosclerosis [86, 87], arthritis [88–90], ureters [91–93], bile ducts [94], and nerves [95, 96]. For visualisation of these fluorescent agents, specialised intraoperative imaging systems have been developed for open surgery [45, 46, 63, 68–70, 97, 98], endoscopy [99], laparoscopy [100], thoracoscopy [101], and robotic surgery [102, 103]. Figure 2 shows the intraoperative impact of using fluorescence-guided surgeries.

Despite a strong driving force coming from the auspicious results of these trials, there is still a fundamental controversy in the scientific field on how to cope with the complex process of optical imaging. Optical properties of the tissue can attenuate fluorescence signals; light scattering, for example, may cause blurred images. In addition, tumour-to-background ratios are influenced by nonspecific autofluorescence of the surrounding healthy tissues, although this disturbance is lower in the NIR region. Further, fluorescence intensity is further dependent on many factors, such as the concentration and fluorescence quantum yield of the fluorescent agent, the affinity of the fluorescent agent, the abundance of target receptors or epitopes, and the imaging system [60]. Innovative calculation methods have been developed that can partly correct for the perturbation caused by tissue optical properties, but a millimetre-scale sharp delineation of the tumour remains challenging in many cases [98, 104–107]. Considering all these factors, sufficient understanding of these processes by the surgeon is essential to adequately interpret the intraoperative fluorescence image at hand.

## Intraoperative use of Raman spectroscopy

Raman spectroscopy is an optical technique that can be used for real-time characterisation of biological tissue in and ex vivo. A Raman spectrum of tissue is a representation of the molecular composition of that tissue. As cancer alters the molecular composition of tissue, RS can be used for discrimination between tumour and healthy tissue. Contrary to FLI, the technique does not require preparation or staining of the tissue to be analysed, which facilitates clinical application. With the use of optical fibres, many body locations can be assessed in vivo using hand-held RS probes [113]. RS has a high diagnostic accuracy for cancer detection, with reported overall diagnostic sensitivities and specificities between 73 and 100% and 66 and 100%, respectively [114]. Its intraoperative application has recently been explored in brain tumour biopsies [115] and brain tumour surgery [116, 117], as well as in debulking procedures of ovarian cancer [118]. RS can further be used for peripheral nerve visualisation and identification and sparing of vital structures during oncological surgery [119, 120].

**Fig. 3:**
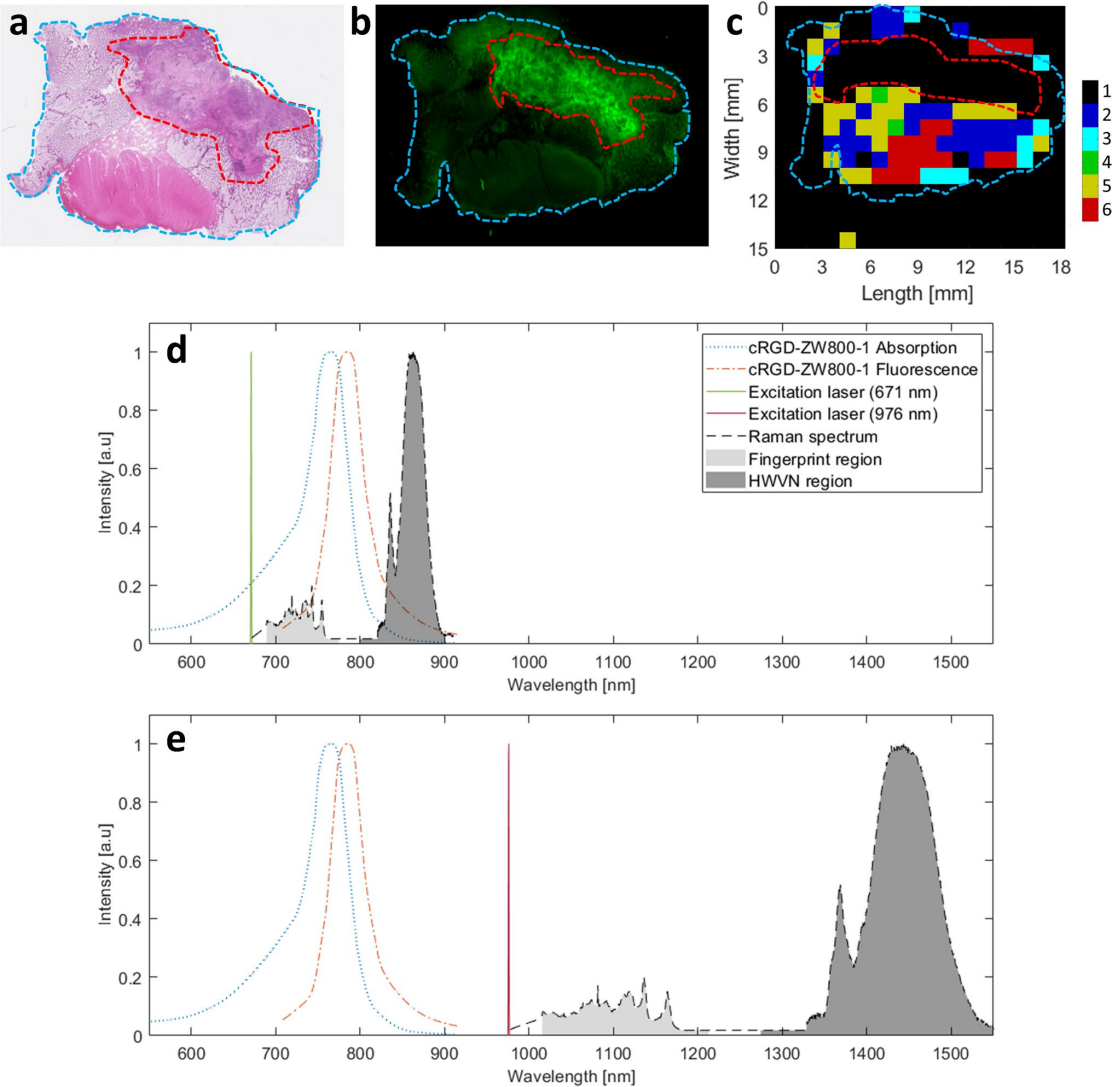
FLI and RS image of tissue section of colorectal cancer containing cRGD-ZW800-1 and surrounding healthy tissue. Ethical approval for collection of this tissue falls under the METC LUMC, as part of studies registered in the European Trials Database under numbers 2016–000,397-38 and 2017–002,772-60 [73]. Tumour is delineated by the red dotted line; tissue is delineated by blue dotted line (a, b, and c). **a** Haematoxylin and eosin–stained slide. **b** Fluorescent image showing colocalisation of signal with tumour. **c** Cluster analysis of the Raman image illustrating that the tumour area cannot be characterised by RS because the fluorescence signal is too strong. **d** Fluorescence spectrum of cRGD-ZW800-1 showing overlap with the acquired Raman signal at an excitation wavelength of 671 nm. HWVN region provides stronger Raman signal intensity than other Raman regions and overlaps with the tail of fluorescence emission. **e** Space between fluorescence spectrum of cRGD-ZW800-1 and the acquired Raman signal at an excitation wavelength of 976 nm

The two regions of the Raman spectrum that are relevant to cancer diagnostics are the fingerprint and the high wavenumber (HWVN) regions (Fig. 3d, e). Most biomedical research on RS studies the fingerprint region (~ 400–2000 cm^−1^). This region is rich in spectral features, including the biochemical signatures associated with lipids, proteins, nucleic acids (DNA), and blood [121]. However, processing times in this region are long; signal intensity is relatively low, and molecular discrimination is generally based on minute spectral differences, necessitating complex and robust machine learning algorithms [61, 122]. Furthermore, in vivo applications for RS require fibre optic probes. These fibres, made of fused silica, generate intense background emission requiring complex optical filtering [123]. Contrary to the fingerprint region, no Raman signal is generated in fused silica in the HWVN region, which ranges from ~ 2400 to 3800 cm^−1^. In addition to its amenability for use with optical fibres, the signal in the HWVN region is much more intense than in the fingerprint region. While it contains less detailed spectral information, the HWVN region can be utilised to distinguish malignant from healthy tissue with a more straightforward approach. An additional benefit of performing RS in the HWVN over the fingerprint region is the greater spectral distance from fluorescence emission, when excited with a red or NIR laser. As a result, the Raman signal in the HWVN region experiences less interference with that of fluorescence as compared to the fingerprint region.

In oral cancer, it has been demonstrated that HWVN Raman spectra can identify cancer ex vivo based on the water concentration in freshly excised specimens [55]. This water concentration differs consistently across the border between tumour and healthy surrounding tissue over a distance of about 5 mm [124]. The water concentration averages from 76% in the tumour to about 54% in the surrounding healthy tissue. The variation in water concentration inside tumour tissue appeared to be much lower (8%) than that in the healthy tissue (24%) [124]. Acquisition of HWVN Raman spectra occurred in a point-based manner, i.e. grid-wise measurements of microscopic tissue volumes with instant signal processing (< 0.1 s).

Based on these findings, the development of an automated fibre optic needle probe for determination of the water concentration profile across the margins of a resection specimen has been initiated. The needle probe is placed on the resection surface and then driven into the tissue while recording spectra at regular distance intervals along its path. The goal is to measure the distance between resection surface and tumour border in this way, to enable rapid and objective margin assessment without the need for specimen grossing [125–127*manuscript in preparation Aaboubout *et al*.*].

The combination of other imaging modalities with selective sampling of suspicious areas by RS as a powerful label-free analytical technique has also been explored [128, 129]. Such approaches include MRI, second harmonic generation, OCT, AFI, total internal reflection fluorescence, or quantitative phase microscopy [130–138]. In general, the basic concept behind these multimodal approaches is to combine the high sensitivity of an imaging modality (i.e. rapid identification of areas that need further inspection) with the high selectivity of RS tissue analysis.

## The potential complementary value of combined fluorescence imaging and Raman spectroscopy

In FLI, the signal originates from the interaction of light with the administrated fluorescent agent binding in the tumour. In RS, however, the signal originates from the interaction of light with the tissue itself, regardless of the specificity of fluorescent agent binding. Owing to the distinctly different physical signal origins of FLI and RS, the provided information is complementary.

FLI allows for real-time whole-field imaging of the surgical field, with high signal intensity. However, fluorescence signal intensity is limited by intrinsic optical tissue properties as well as the concentration and fluorescent quantum yield of the fluorescent agent [60]. On the other hand, RS can detect tumour with uniquely high specificity, but the signal intensity is very weak and can be hindered by photon shot noise originating from light interaction with components of the specimen present in the cross-section of the illumination and detection paths [139]. Additionally, its limitation to point measurements does not allow for real-time, whole-field tissue analysis. Therefore, a combination of FLI that has high sensitivity at the expense of a somewhat lower specificity, with RS that provides high specificity would be very advantageous. FLI would be employed in vivo for real-time identification of fluorescence, followed by objective verification of malignancy with RS to yield superior diagnostic accuracy over either modality used in isolation.

Other than helping to distinguish malignant from healthy tissue (i.e. during excision with tumour-free margins and debulking procedures), FLI has additional advantages for surgical oncology. Due to its ability to scan large surfaces, FLI is a useful tool for identifying additional occult lesions as well as relevant vital structures that require preservation during cancer surgery [140]. FLI has been employed to identify separately located clinically occult tumours in a variety of contexts, including peritoneal metastases [141], sentinel lymph nodes [142], pulmonary [143], and abdominal [144] lesions. Such findings have been described to result in improved survival rates, changes in personalised treatment, and reduced morbidity associated with damage to healthy surrounding structures.

Besides securing adequate margins and achieving conservative resection during cancer surgery, RS can complement FLI in several other ways. When fluorescent areas are detected in the wound bed or elsewhere in the scanned surface (e.g. abdomen), RS could be the ideal tool to instantly assess this suspect region, thereby confirming or ruling out the presence of occult lesions. For unanticipated intraoperative findings such as the detection of clinically occult peritoneal metastases during colorectal cancer surgery [141], subsequent in vivo RS could be used to confirm malignancy without the need for time-consuming fresh frozen sectioning. Even in case of a clinically suspicious non-fluorescent lesion, RS can provide timely analysis. Similarly, RS has potential for use as an additional confirmation of fluorescent findings during debulking procedures and in the identification of vital structures.

However, laser-induced fluorescence itself can be a limiting factor for Raman measurements. Because fluorescent signals are generally orders of magnitude stronger, obscuring the Raman signal, these techniques were traditionally considered mutually exclusive. Figure 3 shows this interference in preliminary tests where RS was performed utilising the standard excitation wavelength of 671 nm on freshly excised tissue of a patient after injection of a fluorescent agent in the NIR-800-nm channel. The interface can be eliminated by shifting the excitation wavelength to 976 nm.

Combining FLI with RS indeed requires careful matching of excitation and emission wavelengths of RS and the exogenous fluorescent agents; several groups have studied various approaches to deal with this issue. The excitation of fluorescent labels can be avoided by shifting the wavelength of the Raman laser to a longer wavelength [145].

In a study using phantoms and breast specimens stained with patent blue dye (BD), it was demonstrated that fluorescence induction from surgical pigments could be circumvented by combining a laser with a longer wavelength (785 nm vs. 685 nm) with an indium gallium arsenide (InGaAs) camera capable of measuring the water/total area ratio of the HWVN spectrum [146]. Noting that haemoglobin and BD have no adverse effect on water content analysis, the Raman system was shown to accurately distinguish tumour from healthy tissue, as well as differentiate between normal and metastatic axillary lymph nodes in the presence of BD [147].

Another study combined fingerprint RS with wide-field AFI spectral imaging for intraoperative margin assessment in breast-conserving surgery [130]. AFI was employed to effectively ‘screen out’ the adipose tissue, enabling RS to more efficiently target the non-adipose tissue regions that are at a higher risk of malignancy, with superior diagnostic value as a result.

When it comes to the combination of FLI and RS, 5-aminolevulinic acid (5-ALA) is the most frequently studied fluorescent agent. In 2006, in vivo fingerprint RS was studied on bladder cancer biopsies obtained under the guidance of 5-ALA-induced protoporphyrin IX (PPIX) FLI [148]. Although a NIR laser emitting at 830 nm was used to reduce the interference of PPIX fluorescence emission in the visible area, additional fluorescence background was observed in the sample obtained from fluorescent areas, obscuring the RS signal and requiring algorithmic removal. Therefore, a new classification algorithm was trained based on biopsies with 5-ALA, which improved accuracy (from sensitivity and specificity of 43% and 71%, to 75% and 89%, respectively) [149]. Furthermore, it was demonstrated how the classification accuracy could be further improved by combining fluorescence and RS information on biopsies with 5-ALA (sensitivity and specificity of 100% and 81%, respectively).

More recently, ex vivo RS was performed to differentiate glioma from normal brain in the presence of 5-ALA [150]. It was shown that RS could identify glioma with an accuracy of 0.85–1.00 (along with a 95% confidence interval) using a 785-nm laser and collecting Raman spectra in the fingerprint region. In optical tissue phantoms, tumour margin could be delineated using this approach [151]. Additionally, fingerprint RS was studied on tissues containing verteporfin and temoporfin, two fluorescent agents (photosensitisers) with activation at ~ 690 and ~ 650 nm, respectively [152]. With fluorescence emission similar to PPIX (600–800 nm), both agents were shown to be compatible with a 785-nm excitation Raman system. However, no clinical in vivo intraoperative studies have been reported yet.

To summarise, several studies showed successful Raman measurements in the presence of fluorescent agents with emission in the visible and NIR-700-nm channel regions. However, to the best of our knowledge, there is no report on performing Raman measurements with the agents in the NIR-800-nm channel (e.g. ICG). ICG is the most widely used fluorescent agent in clinical trials of fluorescence-guided surgery (FGS) [43, 153]. It has peak fluorescence emission in the NIR-I region and a long emission tail that extends into the NIR-II (1000–1700 nm) region [154], which is very challenging to combine with RS because of reduced Raman scattering at higher wavelengths, as well as the limited quantum efficiency of silicon-based CCD detectors, which declines to below 30% beyond 1000 nm.

A novel HWVN RS system was recently developed to characterise tumour in highly pigmented skin lesions (i.e. melanomas) [155–157]. These melanocytic lesions have absorption characteristics at long wavelengths, similar to NIR fluorophores. Tissue characterisation was still feasible after increasing the laser excitation wavelen161. gth to/beyond the NIR region (> 900 nm), demonstrating that the combined and simultaneous use of FGS with NIR fluorophores and RS is technically feasible. Using this HWVN RS system, resection margins can be improved by utilising the optimal characteristics of FLI in the NIR region with tumour-specific fluorescent agents, combined with the specificity of RS based on differences in water concentration in this region. A laser excitation wavelength of 976 nm in combination with a novel low-noise InGaAs detector showed perfect elimination of laser-induced fluorescence of pigmented tissues. Collection of Raman signal in the short-wave infrared (SWIR) region with a high quantum efficiency (> 90%) up to 1570 nm showed minimal fluorescence contribution in the obtained spectra excited with 976-nm laser. RS with a low-noise InGaAs multichannel detector in the SWIR region can open up possibilities of combining RS and FLI in a clinical, intraoperative setting, even in the presence of fluorescent agents in the NIR-800-nm channel.

Although RS is generally a label-free method, systemic injection of molecular imaging agents for Raman signal enhancement has recently been studied preclinically for margin assessment purposes. By selectively accumulating in the tumour tissue, multi-purpose nanoparticles could be used for FLI while simultaneously enhancing the Raman signal, as well as providing the option of photothermal tumour ablation [158–160]. This promising approach still awaits clinical translation.

## Conclusion and future work

While combining FLI with RS to achieve both whole-field imaging of the surgical field and objective margin verification would be very advantageous, these techniques have traditionally been considered mutually exclusive. Careful matching of RS excitation wavelengths and fluorophore emission wavelengths is required, presenting various technical hurdles. This issue could be solved by quantification of differences in water concentration in the HWVN region. After FLI with tumour-specific fluorescent agents, HWVN RS may be used to assess margin adequacy. In addition, evidence for the utility of RS in the identification of occult lesions and vital structures underlines its potential as an adjunct to FGS in all its basic applications. It is worth mentioning that intraoperative imaging techniques can also be beneficial for other applications, not limited to cancer surgery. These include delineation of targeted borders for precise surgical resections, evaluation of vascular perfusion, and identification of vital structures, such as nerves or ureters [140]. The synergistic effect of FLI and RS could also be exploited for these applications.
